# Improving the Cost-efficiency of Preventive Chemotherapy: Impact of New Diagnostics on Stopping Decisions for Control of Schistosomiasis

**DOI:** 10.1093/cid/ciae020

**Published:** 2024-04-25

**Authors:** Luc E Coffeng, Matthew Graham, Raiha Browning, Klodeta Kura, Peter J Diggle, Matthew Denwood, Graham F Medley, Roy M Anderson, Sake J de Vlas

**Affiliations:** Department of Public Health, Erasmus Medical Center, University Medical Center Rotterdam, The Netherlands; Big Data Institute, Li Ka Shing Centre for Health Information and Discovery, University of Oxford; Department of Statistics, University of Warwick; London Centre for Neglected Tropical Disease Research, School of Public Health, Imperial College London; Medical Research Council Centre for Global Infectious Disease Analysis, Department of Infectious Disease Epidemiology, School of Public Health, Imperial College London; Centre for Health Informatics, Computing, and Statistics, Lancaster University Medical School, United Kingdom; Department of Veterinary and Animal Sciences, University of Copenhagen, Denmark; Department of Global Health and Development, Faculty of Public Health and Policy, London School of Hygiene and Tropical Medicine, United Kingdom; London Centre for Neglected Tropical Disease Research, School of Public Health, Imperial College London; Medical Research Council Centre for Global Infectious Disease Analysis, Department of Infectious Disease Epidemiology, School of Public Health, Imperial College London; Department of Public Health, Erasmus Medical Center, University Medical Center Rotterdam, The Netherlands

**Keywords:** bilharzia, fecal smear, cost-efficiency, diagnostic performance, decision criterion

## Abstract

**Background:**

Control of schistosomiasis (SCH) relies on the regular distribution of preventive chemotherapy (PC) over many years. For the sake of sustainable SCH control, a decision must be made at some stage to scale down or stop PC. These “stopping decisions” are based on population surveys that assess whether infection levels are sufficiently low. However, the limited sensitivity of the currently used diagnostic (Kato-Katz [KK]) to detect low-intensity infections is a concern. Therefore, the use of new, more sensitive, molecular diagnostics has been proposed.

**Methods:**

Through statistical analysis of *Schistosoma mansoni* egg counts collected from Burundi and a simulation study using an established transmission model for schistosomiasis, we investigated the extent to which more sensitive diagnostics can improve decision making regarding stopping or continuing PC for the control of *S. mansoni*.

**Results:**

We found that KK-based strategies perform reasonably well for determining when to stop PC at a local scale. Use of more sensitive diagnostics leads to a marginally improved health impact (person-years lived with heavy infection) and comes at a cost of continuing PC for longer (up to around 3 years), unless the decision threshold for stopping PC is adapted upward. However, if this threshold is set too high, PC may be stopped prematurely, resulting in a rebound of infection levels and disease burden (+45% person-years of heavy infection).

**Conclusions:**

We conclude that the potential value of more sensitive diagnostics lies more in the reduction of survey-related costs than in the direct health impact of improved parasite control.

## Teaser

Compared to Kato-Katz fecal thick smears, model-based analyses suggest that the use of more sensitive tests only marginally changes the impact of decisions to locally stop preventive chemotherapy against schistosomiasis in terms of disease burden (person-years lived with heavy infection).

Neglected tropical diseases (NTDs) are a group of diseases that cause a significant health and socioeconomic burden that mostly impacts the poorest parts of the world [1]. Preventive chemotherapy (PC) is a central component in the control and elimination of several NTDs, including trachoma and helminth infections like schistosomiasis [2]. PC constitutes the blanket treatment of populations in endemic areas, regardless of individuals’ infection status. With regular distribution and high enough population coverage, PC is effective at reducing infection levels and morbidity in endemic populations. PC may even lead to complete local interruption of transmission, although sustainable large-scale elimination should also be expected to require behavioral and structural interventions such as improved access to water, sanitation, and hygiene [3, 4]. For the sustainability of NTD control, guidelines by the World Health Organization (WHO) recommend that PC is scaled down or stopped when infection levels in target populations have decreased to sufficiently low levels. This is also the case for the disease schistosomiasis (SCH), which is caused by a parasitic helminth infection that is currently primarily controlled via school-based PC with the drug praziquantel.

SCH is transmitted via contamination of freshwater bodies with human feces or urine and intermediate freshwater snail hosts that release cercarial infective stages [5]. In Africa, the majority of human SCH infections are caused by 2 species, where the adult male-female worm pairs either reside in the venules of the intestines (*Schistosoma mansoni*) or the bladder (*Schistosoma haematobium*). In endemic areas, infection levels typically peak in school-aged children (SAC), although adults can also harbor a substantial fraction of the worm population [6, 7]. The distribution of adult worms across the human population is highly overdispersed, meaning that a few individuals are infected with many worms (up to hundreds or thousands [8]) but most individuals carry only a few or no worms [5]. As a result, a single treatment with praziquantel, which kills approximately 86% of the adult worms [9], is unlikely to eliminate infection in individuals with higher infection intensities. These individuals, along with untreated individuals, are important reservoirs of infection from which transmission continues between PC rounds. Therefore, PC must be implemented repeatedly and at sufficiently high coverage levels in order to successfully reduce infection to low levels [10].

An increasing number of SCH-endemic areas are approaching a point where, after 5 to 6 years of PC, it may be possible to scale down or even stop PC if the prevalence of infection in SAC is low enough (eg, 2%) [11]. The WHO-recommended diagnostic technique to measure infection levels is microscopy-based detection of SCH eggs by either Kato-Katz (KK) fecal smears or urine filtration, or a point-of-care circulating cathodic antigen test (*S. mansoni* only) [12]. However, an often-raised concern is that egg detection methods are suboptimal due to their poor sensitivity to detect low-intensity infections [13–15], which constitute the majority of infections, especially after repeated PC rounds. The concern is that decisions based on low-sensitivity tests may lead to prematurely stopping PC and that infection levels will quickly bounce back. Another concern is that when population infection levels are very low, the cost of a survey based on the currently recommended diagnostics may be higher (due to required high sample sizes) in some settings than the delivery of a round of PC itself [13]. It has therefore been suggested that policy decisions should be based on more sensitive new molecular tests [16]. And ideally, surveys employing such new tests should be performed as a single field visit, and the cost of the assessment should be less than the cost of 2–3 rounds of PC [14, 15].

In this study, we investigate the extent to which the use of more sensitive diagnostic techniques can contribute to improved decision making for PC. We hypothesize that the use of better diagnostic techniques may improve the identification of populations that do and do not need PC, leading to the same or improved health impact, possibly with fewer PC treatments distributed. Here, we focus on populations that are endemic for *S. mansoni* (intestinal SCH) where PC is targeted at SAC. We employ an existing SCH transmission model [17–20] to simulate the impact of PC and the outcome of different diagnostics strategies to inform stopping decisions for PC. To accurately capture how the sensitivity of KK changes with individual worm burden, we analyzed historical data representing 7 days of duplicate *S. mansoni* egg counts from Burundi [21]. We then compared the performance of decision strategies based on single and duplicate KK, as well as a range of new hypothetical diagnostic tests with higher sensitivity for detection of low-intensity infections.

## METHODS

### Mathematical Model for Trends of *S. mansoni* Infection During and After PC

We employed a published previously age-structured individual-based stochastic model [17–20]. The model assumes that *S. mansoni* worms are monogamous and that their distribution has a negative binomial form. The within-host section of the model describes the evolution of the worm burden in individuals as a function of age. The model was implemented in Python, for which the code can be found at: https://github.com/NTD-Modelling-Consortium/ntd-model-sch/tree/Endgame_v2 . A mathematical description of a deterministic version of the model is provided in the Supplementary Data. There, we also describe how we adhered to the PRIME-NTD principles (Policy-Relevant Items for Reporting Models in Epidemiology of Neglected Tropical Diseases) [22].

The model was expanded with mechanisms to dynamically stop PC during the simulation, conditional on a survey result being under a user-defined threshold. Furthermore, to better capture the sensitivity of KK variants, we updated model concepts for simulation of egg counts by adding structured variation of egg counts by day and by repeated slide, which were governed by 2 shape parameters $k_{day}$ and $k_{slide}$ (lower values indicate higher variation and values k≥5 are suggestive of little evidence for overdispersion; for technical details, see Supplementary Data). We further added model concepts for new hypothetical diagnostic tests that can detect individual worms. To capture that the sensitivity of such tests increases with the intensity of infection, we defined sensitivity $S_{t}$ of test *t* as a function of the number of adult worms *N* and the probability $P_{t}$ that the test can detect a single worm. This means that $1-P_{t}$ is the probability that a worm will escape detection and that $(1-P_{t}{)}^{N}$ is the probability that all *N* worms will escape detection. Therefore, overall test sensitivity was defined as $S_{t}=1-(1-P_{t}{)}^{N}$.

### Quantification of Diagnostic Variation in Fecal Egg Counts Based on Kato-Katz

To quantify the 2 shape parameters $k_{day}$ and $k_{slide}$ that govern variation in individuals’ fecal egg counts by day and slide, we analyzed a historical dataset comprising 7 days of duplicate *S. mansoni* egg counts from 200 individuals in Burundi [21]. These data were based on KK slides of 1/40 = 0.025 gram feces, which deviates from the recommended 1/24 = 0.417 gram that is typically used nowadays. Egg counts were modeled using a Bayesian statistical model, assuming that counts follow an overdispersed Poisson distribution that captures variation between individuals, between days, and between repeated slides based on the same fecal sample [23] (for technical details, see Supplementary Data).

### Simulating the Impact of Different Diagnostic Strategies for Decisions to Stop PC

Simulations were run for a population of 3000 humans, which was taken to represent a small homogenous transmission area. Transmission conditions in terms of the basic reproductive number *R_0_* (recording the average transmission level in the population) and a shape parameter *k* (individual exposure heterogeneity) were allowed to vary randomly between repeated simulations such that the baseline prevalence of infection in SAC was between 0% and 25% (based on single KK). For each set of transmission conditions, we simulated 5 years of PC targeting SAC at 90% coverage. One year after the fifth PC round, just prior to a sixth round, a survey was simulated that tested 450 SAC (about half of SAC) with one of various diagnostic strategies (details below). If the survey resulted in a prevalence estimate under the user-defined threshold, PC was automatically stopped after the sixth PC round, and if not, PC was continued and another survey was done 4 years later. Surveys were simulated every 4 years until a decision to stop PC was reached.

Decisions to stop PC were based on either single KK, duplicate KK (2 slides based on the same fecal sample), or 1 of 6 hypothetical new diagnostic tests that were characterized in terms of test sensitivity and specificity. The sensitivity of the new diagnostic tests was formulated as a per-worm-probability $P_{t}$ to be detected. Values of $P_{t}$ were chosen such that the lowest value corresponded to the sensitivity of duplicate KK to detect infections with 1–2 worm pairs (assuming each worm pair contributes *α* = 0.34 eggs to a single KK of 1/24 gram, as in the transmission model). The specificity of new diagnostic tests was assumed to be 99% or 100%; the specificity of KK was assumed to be 100%. To stop PC, we considered 3 thresholds for the measured infection prevalence in SAC (1%, 2%, or 5%), which were chosen for illustrative purposes as in an earlier modeling study [11]. Simulations were performed for 200 random transmission conditions, and for each of these, simulations were repeated 3 times (with different random number seeds) for a total of 600 repeated simulations per diagnostic strategy. Simulations with a baseline prevalence of 0% were discarded.

## RESULTS

### Model Quantification for Sensitivity of Kato-Katz and New Hypothetical Diagnostic Tests

**Figure 1.**
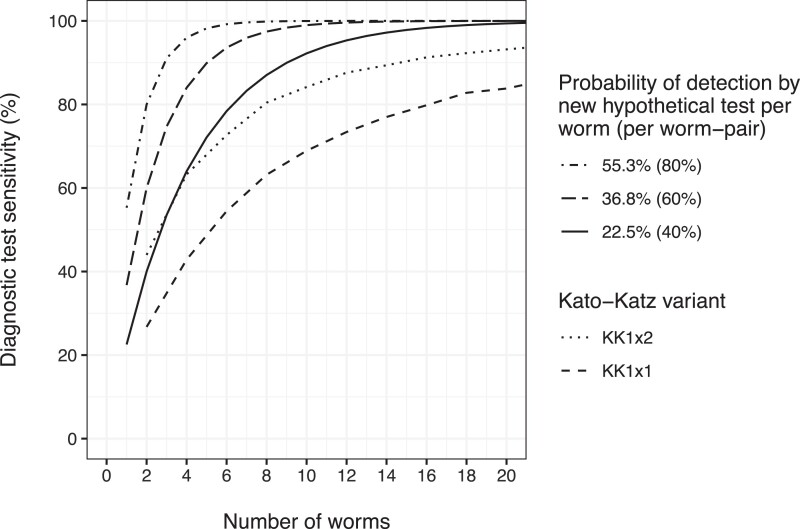
Estimated sensitivity of diagnostic tests as a function of the number of adult worms. The estimated sensitivity of Kato-Katz (KK) is conditioned on the point estimate for variation in egg counts by day ($k_{day}=1.68$) and slide ($k_{slide}=2.37\cdot \frac{40}{24}=3.95$, representing slides based on 1/24 gram of feces), and the assumption that, on average, each worm pair contributes α=0.34 eggs to a single KK of 1/24 gram, as in the individual-based stochastic model. KK1×1 and KK1×2 indicate single and duplicate KK slides, respectively.

Based on the Burundian KK data, we estimated that within-individual variation in egg counts by day and slide was considerable, with the shape parameters of the corresponding gamma distributions estimated at $k_{day}=1.68\;(95\text{\%}\text{-}\;\mathrm{Bayesian}$  credible interval:1.40−2.03) and $k_{slide}=2.37\;(95\text{\%}\text{-}\;\mathrm{BCI}:\;$  1.99−2.82). This means that variation in egg counts was driven more so by temporal sources (lower *k*) than slide-by-slide variation (higher *k*). As the data were based on 1/40 grams of feces per KK slide, we translated our estimate of $k_{slide}$ to a value $k_{slide}^{\prime }$ for KK slides based on 1/24 grams of feces by setting $k_{slide}^{\prime }=k_{slide}\cdot \frac{40}{24}=3.95$. Together, $k_{day}$ and $k_{slide}^{\prime }$ translated to a coefficient of variation of individual-level egg counts of 1.00(95%-BCI:.94−1.07). The sensitivity of single and duplicate KK tests (based on the same fecal sample) is shown for a range of worm burdens in Figure 1.

In case of 2 to 4 adult worms (or the equivalent of 1 to 2 worm pairs), the new hypothetical diagnostic test with low sensitivity (*P_t_* = 22.5%, ie, 40% per worm pair, solid line in Figure 1) performed similarly to a duplicate KK (black dotted line). Between 4 and 20 adult worms (2–10 worm pairs), the sensitivity of this test approached 100%, whereas the sensitivity of duplicate KK only reached around 95% for 10 worm pairs. Therefore, for our simulations of different diagnostic strategies, we adopted 3 values of *P_t_* for new hypothetical diagnostic tests: 22.5% (low), 36.8% (moderate), and 55.3% (high), which correspond to a 40%, 60%, and 80% probability of detection per worm pair.

### Simulating the Impact of Different Diagnostic Strategies for Decisions to Stop PC

**Figure 2.**
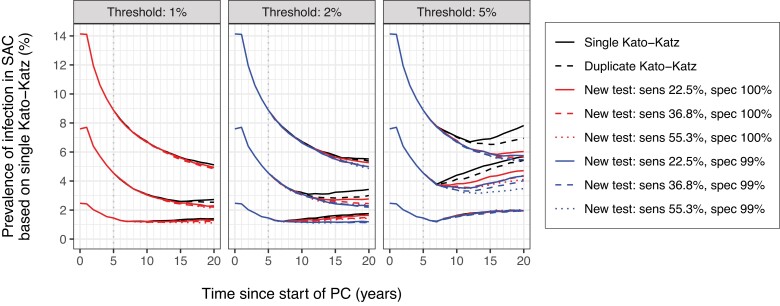
Model-predicted average trends of *Schistosoma mansoni* infection in school-aged children (SAC) under different diagnostic strategies for the decision to stop preventive chemotherapy (PC). The 3 panels pertain to different prevalence thresholds (1%, 2%, and 5%) for making the decision to stop PC. Trend lines represent averages over repeated simulations across 3 categories of baseline prevalence in SAC (<5%, 5%–10%, and 10%–25%). Note that for the scenario with 1% decision threshold (left panel), tests with 99% specificity were not simulated as these were considered incompatible with the threshold. PC was assumed to be implemented annually at 90% coverage of SAC. Abbreviations: sens, sensitivity; spec, specificity; PC, preventive chemotherapy; SAC, school-aged children.

We next predicted infection trends under different diagnostic strategies, based on 546 of 600 simulations for which the baseline infection prevalence was >0% (Supplementary Figure 1). If the decision to stop PC was based on KK as a diagnostic technique, the expected infection trends were very similar for single and duplicate KK (Figure 2, black lines). Furthermore, if using a new hypothetical test with sensitivity for low worm burdens similar to KK ( $P_{t}=22.5\text{\%}$), the predicted trends were very similar to when using KK itself (red solid lines). For a 1% decision threshold, more sensitive diagnostic tests only marginally changed expected trends (red dashed and dotted lines in left panel). For the 2% threshold, tests that were more sensitive (dashed and dotted lines) or less specific (blue lines) resulted in the decision to stop PC to be made later, on average, leading to slightly lower infection prevalence. For the 2% threshold and settings with a baseline prevalence of 5%–10%, infection levels visibly rebounded, although slightly less markedly so for the more sensitive and less specific diagnostic tests. The same was the case for the 5% threshold (right panel).

**Figure 3.**
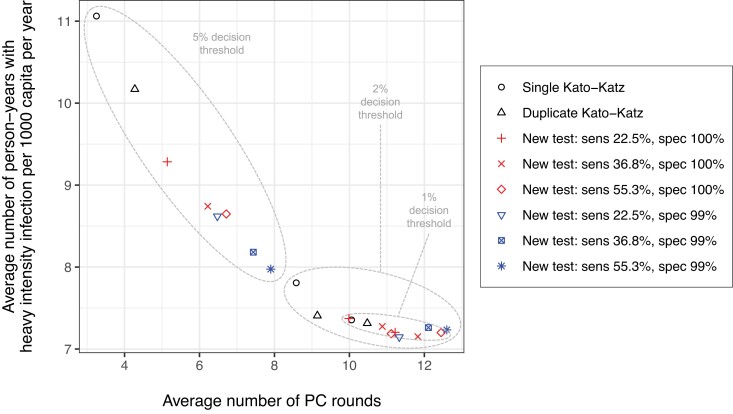
Model-predicted person-years with heavy *Schistosoma mansoni* infection in the general population versus the number of rounds of school-based preventive chemotherapy (PC) under different diagnostic strategies for the decision to stop PC. PC was assumed to be implemented annually at 90% coverage of school-aged children (SAC). Symbols and colors represent different diagnostic strategies to make decisions about stopping PC. Person-years with heavy infection and number of PC rounds were calculated only for the last 15 years of the simulation, that is, from the point of the first survey at year 5 onward and after the sixth PC round had taken place. This means that at most, 14 PC rounds could have been delivered after the first survey. The result shown here represent settings with baseline prevalence of infection in SAC of 5%–10%. For results in other baseline prevalence settings, see Supplementary Figure 2. Abbreviations: sens, sensitivity; spec, specificity; PC, preventive chemotherapy.

To assess the impact of different diagnostic strategies for decisions to stop PC, we quantified the disease burden in terms of the average number of person-years with heavy intensity infections (all ages) from the start of the first survey (year 5) till 15 years later, and compared this to the average number of PC rounds that was distributed during the same period. In Figure 3, we see that for settings with a baseline prevalence of 5%–10%, stopping PC based on single KK and a threshold of 2% resulted in about 9.5 person-years of heavy infection per 1000 capita per year and distribution of on average 11.0 PC rounds. For most diagnostic strategies, a less stringent threshold of 5% resulted in fewer PC rounds (4.3 up to 11.1, depending on the diagnostic used), but came at the cost of a higher disease burden (up to 12.6 person-years of heavy infection per 1000 capita per year). The exception was the highest sensitive test with 99% specificity, which performed very similar to single KK combined with a 2% threshold. More conservative diagnostic strategies (a 1% threshold or use of more sensitive or less specific tests) led to a comparable or slightly lower disease burden, but at the cost of slightly more PC rounds. For the other 2 baseline prevalence categories, these patterns were qualitatively similar (Supplementary Figure 2). The probability of achieving 0% prevalence of infection within 20 years from the start of PC was 0% for settings with a baseline prevalence in SAC of ≥5%, regardless of diagnostic strategy. Only for settings with a baseline prevalence in SAC of <5%, the probability was between 4% and 6% (Supplementary Figure 3) with only small differences between diagnostic strategies.

## DISCUSSION

We illustrate that for local decisions to stop PC, the currently recommended diagnostic technique, KK, using a 2% decision threshold, almost fully minimizes the disease burden (in terms of person-years with heavy infection) with the lowest possible number of PC rounds to reach that impact. New diagnostics with higher sensitivity for detection of low-intensity infections may lead to a marginally lower disease burden, but at the cost of additional PC rounds. The same outcome can also be achieved by lowering the decision threshold for KK-based decisions to 1%. If decision thresholds for surveys based on new improved tests are adjusted upward (here to 5%) to account for higher test sensitivity, there is a risk of prematurely stopping PC. In the real world, this would mean that PC would have to be restarted (for which we do not consider the cost here); without PC, infection levels would likely bounce back, and via human mobility could even lead to reintroduction of infection in areas where transmission was previously interrupted. This aspect of reintroduction is currently not considered in existing SCH transmission models, which do not include a spatial component.

Our findings support the recently updated WHO guidelines for SCH control, which conclude that there is good evidence to support the continued use of KK to detect *S. mansoni* in the context of PC programs [12]. These guidelines further support the use of point-of-care circulating cathodic antigen tests, which are far more sensitive than KK [24]. We show that if such more sensitive tests are used to inform policy decisions, a higher prevalence criterion should be used to avoid unnecessarily long continuation of PC. This is especially relevant as the WHO diagnostic technical advisory group stated that the cost of a survey should be less than the cost of 2–3 rounds of MDA [14, 15]. However, diagnostic-specific decision criteria remain to be developed. This will require more in-depth analyses of the differences and correlation between results from KK and alternative diagnostic tests such as circulating cathodic antigen tests, and what survey designs are most cost-efficient for decision making (eg, what age groups to test, how many, and when).

This study was inspired by the notion that more sensitive diagnostic tests might contribute to more accurate decisions to stop or continue PC by improving the identification of areas that do and do not need PC [16]. Hypothetically, this would lead to the same or higher health impact with possibly fewer PC treatments distributed. However, if making local decisions about PC, we found that there is little to gain in terms of health impact by the use of new diagnostics. In fact, given the high efficacy of praziquantel, the health impact of PC against schistosomiasis is primarily driven by which age groups are targeted and the achieved population coverage [7, 25]. As for the accuracy of decisions to stop or continue PC, more sensitive tests (when combined with an appropriate decision threshold) should be expected to increase the probability of achieving elimination after stopping PC, although our simulation study was not powered enough to demonstrate this. In general, decision accuracy is mainly determined by pre-PC infection levels, the survey design (selection of study sites and age groups), the sample size (number of sites and number of persons per site), and the decision criterion for stopping PC [11, 26–28]. Previous work has shown that the use of more sensitive diagnostic tests may allow for smaller sample sizes to achieve similar levels of accuracy in decision making, thus potentially saving costs [27]. A fair comparison of diagnostic tests with regard to the trade-off of cost and accuracy of policy decisions would require detailed cost data and information on the diagnostic variability of each diagnostic [29].

In this study, we simulated decisions to stop PC for relatively small areas (∼3000 people) and did not consider that currently, decisions are made for larger areas where the distribution of SCH may be highly focal. Given this focality and the finite stock of praziquantel, it has been suggested that more focalized PC distribution and decision making may be warranted [16]. For more focal decision making, new diagnostics may well be more cost-efficient than Kato-Katz because of feasibility of implementation (eg, a point-of-care lateral flow test) or lower cost per test. Quantification of these potential benefits would require either of 2 approaches. The first is to explicitly model transmission of infection and mobility of humans in larger geographically heterogeneous areas, for which initial attempts have been made for onchocerciasis [30, 31], another helminth targeted with PC. The second approach would be a Monte Carlo simulation study of the cost and performance of spatial survey designs. Such a study would have to be informed by data on spatial heterogeneity in prevalences collected in an area where the average prevalence is around the decision threshold after multiple years of PC, analyzed with either mixed effects models [32] or geospatial models.

In this study we did not explicitly consider costs of PC, diagnostics, or consequences of suboptimal policy decisions. To better inform and design cost-efficient policies and decision strategies for the control of SCH, but also NTDs in general, there is an urgent need for estimates of the cost of making a “wrong decision.” What are the costs of continuing PC for too long, or the costs of stopping PC too early? The first is relatively straightforward and may be captured well enough by simply counting the cost per PC rounds or distributed treatment. The second, however, is much more challenging as from a program or societal perspective, in addition to the “saved cost” on PC rounds, one should also capture the cost of having to restart PC, which may require renewed investments. Hopefully, such investments are limited as national NTD control programs have matured over the last decade and are becoming more and more integrated across NTDs, meaning that stopping PC against 1 NTD may be less likely to lead to loss of expertise and infrastructure. However, this is speculation, and it is probably safer to assume that we would rather implement a few PC rounds too many than to stop PC too early.

In conclusion, we illustrate that compared to KK, the use of more sensitive tests for decisions to stop or continue PC at a local level will at best only marginally improve the health impact of PC programs against SCH, at the cost of potentially implementing additional PC rounds. However, more sensitive diagnostic tests may still help to correctly identify the last cases of infection and to improve the cost-efficiency of SCH control via lower cost per survey and improved feasibility of conducting surveys locally.

## Supplementary Data

Supplementary materials are available at *Clinical Infectious Diseases* online. Consisting of data provided by the authors to benefit the reader, the posted materials are not copyedited and are the sole responsibility of the authors, so questions or comments should be addressed to the corresponding author.

## Supplementary Material

ciae020_Supplementary_Data

## Notes

***Author contributions.*** L. E. C. conceptualized and performed the statistical analyses of the Burundian data, conceptualized the transmission model components for simulation of Kato-Katz and new hypothetical tests, analyzed and visualized the data, and drafted the manuscript. M. G. performed the simulations with the transmission model, analyzed and visualized the simulation output, and revised the manuscript. R. B., K. K., R. M. A., M. D., G. F. M., and S. J. d. V. interpreted the data and revised the manuscript. P. J. D. interpreted the data, verified that the Burundian data did not contain residual temporal correlation that might not have been captured by the statistical model, and revised the manuscript.

***Acknowledgments.*** We gratefully acknowledge Amanda Minter for her coordination of the project with the Endgame team at the Bill & Melinda Gates Foundation. We also gratefully acknowledge Dr Dirk Engels for the original collection of the Burundian egg count data used in this study.

***Disclaimer.*** The funders had no role in study design, data collection and analysis, decision to publish, or preparation of the manuscript.

***Financial support.*** This work was supported by the Bill & Melinda Gates Foundation (INV-030046), via the Neglected Tropical Diseases (NTD) Modelling Consortium. K. K. and R. M. A. also acknowledge funding from the Medical Research Council Centre for Global Infectious Disease Analysis (MR/R015600/1), jointly funded by the UK Medical Research Council (MRC) and the UK Foreign, Commonwealth and Development Office (FCDO), under the MRC/FCDO Concordat agreement, and is also part of the EDCTP2 Programme supported by the European Union.

***Supplement sponsorship.*** This article appears as part of the supplement “New Tools and Nuanced Interventions to Accelerate Achievement of 2030 Roadmap for Neglected Tropical Diseases,” sponsored by funding of Professor T. Déirdre Hollingsworth's research by the Li Ka Shing Foundation at the Big Data Institute, Li Ka Shing Centre for Health Information and Discovery, University of Oxford; and funding of the NTD Modelling Consortium by the Bill & Melinda Gates Foundation (INV-030046).
