# Supplementary material for: Improving the Cost-efficiency of Preventive Chemotherapy: Impact of New Diagnostics on Stopping Decisions for Control of Schistosomiasis
Source: Clin Infect Dis. 2024 Apr 25;78(Suppl 2):S153–9. doi: 10.1093/cid/ciae020 (PMC11045014; doi:10.1093/cid/ciae020)
Supplement: ciae020_Supplementary_Data [file ciae020_supplementary_data.pdf]

# Supplementary Data to "Improving the cost efficiency of preventive chemotherapy: impact of new diagnostics on stopping decisions for control of schistosomiasis"

Luc E. Coffeng[a*], Matthew Graham[b], Raiha Browning[c], Klodeta Kura[d,e], Peter J. Diggle[f], Matthew Denwood[g], Graham F. Medley[h], Roy M Anderson[d,e], Sake J. de Vlas[a]

[a] Department of Public Health, Erasmus MC, University Medical Center Rotterdam, Rotterdam, The Netherlands

[b] Big Data Institute, Li Ka Shing Centre for Health Information and Discovery, University of Oxford, Oxford, United Kingdom

[c] Department of Statistics, University of Warwick, United Kingdom

[d] London Centre for Neglected Tropical Disease Research, Department of Infectious Disease Epidemiology, School of Public Health, Imperial College London, London, United Kingdom

[e] MRC Centre for Global Infectious Disease Analysis, Department of Infectious Disease Epidemiology, School of Public Health, Imperial College London, London, United Kingdom

[f] CHICAS, Lancaster University Medical School, Lancaster, United Kingdom

[g] Department of Veterinary and Animal Sciences, University of Copenhagen, Denmark

[h] Department of Global Health and Development, Faculty of Public Health and Policy, London School of Hygiene and Tropical Medicine, London, United Kingdom

* Corresponding author: l.coffeng@erasmusmc.nl; postal address: Erasmus MC, Department of Public Health, P.O. box 2040, 3000 CA Rotterdam, The Netherlands.

## Supplementary Data

### Model for *Schistosoma mansoni* transmission

The model used to describe the worm burden of individuals of a given age and the quantity of infectious eggs in the environment was developed from the founding work of Anderson and May [1]. Importantly, we assume that the dynamical timescale of the miracidia, cercaria and snail intermediate host, i.e., the reciprocal of their typical respective life spans, are all very fast relative to the life span of the adult worms in the human host (hours and weeks compared with years for the adult worms). This allows us to collapse the equations describing the dynamics of these stages into the equation representing changes in adult worm load within the human host [2]. Briefly, the model is a stochastic individual-based implementation of the original partial differential equation (PDE) model describing the evolution of mean female worm burden as a function of age, $M(a,t)$:

$$\frac{\partial M(a,t)}{\partial t}+\frac{\partial M(a,t)}{\partial a}=L\beta(a)-\sigma M(a,t)$$

Here, $L$ is the concentration of infectious material in the environment. The model describes the evolution of the female worm burden and assumes they are distributed according to an underlying negative binomial distribution. The dynamics of infectious material is governed by

$$\frac{dL}{dt}=\psi\lambda\int_{a=0}^{\infty}[M(a)\cdot f(M(a),z,k)\cdot\rho(a)\cdot P(a)]da-\mu_2 L$$

Where.$P(a)$ is the normalised age distribution for the population. The function $f(.)$ describes the production of fertile infectious material and is the product of a term representing the dampening effect of density dependent fecundity at higher worm burdens [first term] and the catalytic effect of the presence of male worms on sexual reproduction at very low worm burdens [second term] [3]:

$$f(M(a),z,k)=\left[1+\frac{(1-z)M(a)}{k}\right]^{-(k+1)}\phi(M(a),k)$$

where $z=e^{-\gamma}$ represents the strength of density-dependent fecundity and $k$ is the negative binomial aggregation parameter, as discussed in the main text. The function $\phi(.)$ approximates the effect of monogamous sexual preproduction on egg production, where

$$\phi(m,k)=1-\frac{(1-\alpha)^{1+k}}{2\pi}\int_0^{2\pi}\frac{(1-\cos(\theta))d\theta}{(1-\alpha\cos(\theta))^{1+k}}$$

where $\alpha=m/(k+m)$. The parameter $\psi$ characterises the flow of infectious material into the environment. This parameter and the absolute magnitude of $\beta$ and $\rho$ are subsumed into the definition of the basic reproduction number, $R_0$, which measures the intensity of the transmission cycle.

$$R_0=\frac{z\lambda\psi}{\mu_2}\int_0^{\infty}\rho(a)P(a)\int_0^{a}\beta(x)e^{-\sigma(a-x)}\,dxda$$

Assuming a 1:1 sex ratio in worms, the total worm burden is given by $2M(a,t)$. Egg counts for individual hosts of age $a$, $E(a)$, can be seen as a component of the contribution of host egg output into the environment

$$E(a)=\lambda M(a)f(M(a),z,k)$$

where $\lambda$ is the female worm fecundity parameter.

## Updated model concepts for diagnostic test sensitivity

We employed an individual-based stochastic model for *Schistosoma mansoni* transmission that was developed and published in previous work [2,4–6]. Originally, in this model, egg counts based on Kato-Katz (KK) are simulated using a negative binomial draw with mean equal to the expected egg count and a single shape parameter governing the level of overdispersion between repeated egg counts. However, as soon as more than one egg count per person and time point is simulated, this approach assumes that repeated egg counts are identically independently distributed, regardless of whether they are based on the same stool sample or not. In contrast, egg counts based on repeated slides prepared from the same stool sample should be expected to be more correlated than repeated counts based on stool samples from different days.

To better capture the sensitivity of KK variants (single vs. duplicate slides based on the same faecal sample) at low levels of infection, we updated the previously used model concepts for simulation of egg counts with structured variation by day and by repeated slide. This was achieved by simulating egg counts from a gamma-gamma-Poisson compound distribution [7], where the two nested gamma distributions capture variation by day and by slide. For each simulated individual $i$, the expectation (mean) of the gamma-gamma-Poisson compound distribution was defined as in the original model [2,4–6] using a density-dependent function $f(.)$ of the individual's worm-pair count $M_i$: $f(M_i) = \lambda \cdot M_i \cdot e^{-z \cdot M_i}$, which captures that with increasing numbers of worm pairs, the number of eggs produced per worm pair decreases. Here, $\lambda = 0.34$ is the expected number of eggs per worm pair per Kato-Katz slide in absence of density dependent fecundity; and $z = 0.0007$ governs the degree of density dependence [6]. The degree of variation by day and slide was governed by the two shape parameters $k_{day}$ and $k_{slide}$ of the two nested gamma distributions, for which values were based on a statistical analysis of field data from Burundi (details in the next section below).

For simulation of surveys based on hypothetical new diagnostic tests, we developed and implemented new model concepts for detection of individual worms. Because the sensitivity of helminth diagnostics typically depends on intensity of infection, we defined sensitivity $S_t$ of test $t$ as a function of the number of adult worms $N$ and the probability $P_t$ that the test can detect a single worm. This means that the $1 - P_t$ is the probability that a worm will escape detection and that $(1 - P_t)^N$ is the probability that all $N$ worms will escape detection. Therefore, we define overall test sensitivity as $S_t = 1 - (1 - P_t)^N$.

## Quantification of diagnostic variation in faecal egg counts based on Kato-Katz

To quantify the two shape parameters $k_{day}$ and $k_{slide}$ that govern variation in individuals' faecal egg counts by day and KK slide, we analysed a historical dataset comprising seven days of duplicate *S. mansoni* egg counts from 200 individuals in Burundi [8]. We note that these data were based on KK slides of $1/40 = 0.025$ gram faeces, which deviates from the recommended $1/24 = 0.417$ gram that is now more typically used (this was accounted for in our analysis). Egg counts were modelled using a Bayesian statistical model, assuming that counts follow an overdispersed Poisson distribution that captures variation between individuals, between days, and between repeated slides based on the same faecal sample. These three variance components (individuals, faecal samples, and slides) were modelled using three compounded gamma distributions (parameterised in terms of shape and rate) and Poisson variation representing the counting variation in the observed egg counts [7]:

$$\mu_i \sim \Gamma\left(k_{between}, \frac{k_{between}}{\mu}\right)$$

$$\mu_{id} \sim \Gamma\left(k_{day}, \frac{k_{day}}{\mu_i}\right)$$

$$\mu_{ids} \sim \Gamma\left(k_{slide} \cdot \frac{w_{sample}}{0.025}, \frac{k_{slide}}{\mu_{id}}\right)$$

$$\text{count}_{ids} \sim Pois(\mu_{ids})$$

Here, $\mu$ represents the average egg count at the population level; $\mu_i$ and $\mu_{id}$ represent the expected egg count for an individual on any day and one particular day, respectively; $\mu_{ids}$ indicates the expected egg count for a single KK for a slide based on $w_{sample}$ grams of faeces. Higher values of $k$ indicate a lower coefficient of variation and therefore less overdispersion. We note that a compound gamma-Poisson distribution has the same distribution function as the negative binomial distribution, such that effectively: $\text{count}_{ids} \sim NB\left(\mu_{ids} \cdot \frac{w_{sample}}{0.025}, k_{slide} \cdot \frac{w_{sample}}{0.025}\right)$, which clearly illustrates how the mean and level of overdispersion of repeated slides based on the same faecal sample change with the amount of faeces used per slide. We further note that while the mean and variance of compound gamma-gamma distributions are simple to calculate [9], the resulting distribution is not itself identical to a gamma distribution, so the overall distribution of egg counts we simulate is not exactly negative binomial.

The contribution of each variance component towards the total variation in egg counts was expressed in terms of the coefficient of variation per component ($CV_{between}$, $CV_{day}$, $CV_{slide}$). For a gamma distribution with shape parameter $k$, this is defined as $CV = k^{-1/2}$ (and conversely, $k = CV^{-2}$ ). The total coefficient of variation over all three levels is $CV_{total} = \sqrt{\prod_i (CV_i^2 + 1) - 1}$. Exploratory analyses showed that specifying marginal prior distributions for individual $CV$ components led to unreasonably thick tails on the push-forward prior for the total coefficient of variation $CV_{total}$, which did not match prior expectations or the data itself. Therefore, we formulated a prior for $CV_{total}$ and a prior for the relative contribution $\kappa_i$ of each individual variance component to $CV_{total}$, such that: $CV_i = \sqrt{(CV_{total}^2 + 1)^{\kappa_i} - 1}$. Here, we satisfy the required condition $(CV_{total}^2 + 1) = \prod_i (CV_{total}^2 + 1)^{\kappa_i}$ by defining $\kappa_{1:3}$ as a simplex vector (i.e., $\sum_i \kappa_i = 1$, where $0 \leq \kappa_i \leq 1$). For $CV_{total}$, we defined a weakly informative half-normal prior distribution $N^+(0, \sigma_{CV0})$, with its standard deviation set to twice the empirical CV of the data ($\sigma_{CV0} = 5.79$). For $\kappa_{1:3}$ we specified a Dirichlet prior with shape 1 for all elements (i.e., $Dir(\alpha_{1:3})$, where $\alpha_i = 1$), which is a uniform prior over the simplex parameter space. For the population average egg count, we defined a weakly informative half-normal prior $N^+(0, \sigma_{\mu 0})$ with its standard deviation set to twice the empirical mean of the data ($\sigma_{\mu 0} = 11.3$).

The model was implemented in Stan [10], and verified by simulating data from the model, and then ensuring that we were able to recover the original input parameters by analysing the simulated data. In addition, the model was also independently implemented and tested in separate software by another author using JAGS [11] in order to further verify the inference. The final analysis was done using the Stan model in R [12] via the package *rstan* [10], using 4 parallel Markov chains and 4000 samples per chain, of which the first 2000 were discarded. In sensitivity analyses, we verified that analysis of data points from only the first three time points (days 1, 3, and 5, so excluding days, 8, 10, 32, and 37) led to the same results as when including data from all days.

**PRIME-NTD table: Policy-Relevant Items for Reporting Models in Epidemiology of Neglected Tropical Diseases**

| Principle | What has been done to satisfy the principle? | Where in the manuscript is this described? |
|---|---|---|
| Stakeholder engagement | The work was initiated following a request from the Bill & Melinda Gates Foundation (BMGF), Seattle. BMGF was consulted twice to clarify the research question and translate it to a model-answerable question. In a third meeting, results were presented to BMGF, and it was concluded that further research is warranted, particularly on the impact of moving from district-level to more local decision-making for PC. Further, this work has been presented at the Annual Meeting of NTD Modelling Consortium (NTDMC) held at Oxford in April, 2023, where representatives from the World Health Organization (WHO) and BMGF were present. | In this table |
| Complete model documentation | We used a previously published model, the details of which are available elsewhere (relevant sources cited in the methods section). Further, we have provided the full description of newly added model components. We further provide a link to an online repository with the Python version of the model. | Methods section and Supplementary Data |
| Complete description of data used | The data relevant for this work were quantified and described in detail in previous publications. These articles are mentioned in the Methods. | Methods section |
| Communicating uncertainty | We have considered parameter in terms of transmission conditions and a range of scenarios for performance of new diagnostics | Methods and Results sections |
| Testable model outcomes | All the model predictions are testable, provided the relevant data are collected (although this may be challenging). | |

**Results of individual simulations**

For each diagnostic strategy we performed 600 repeated simulations, which were based on 200 different parameter sets for transmission conditions and 3 repeated simulations per parameter set. This was done by first running one 5-year-long simulation for each of the 200 transmission conditions and saving the final state of the population. Then, for each diagnostic strategy, we ran 15 years of dynamic PC policy (as described in the methods in the main text), reusing the same 200 saved population states as the initial state. These last 15 years were simulated in triplicate with different random number seeds, leading to a total of 600 simulated infection trajectories per diagnostic strategy.

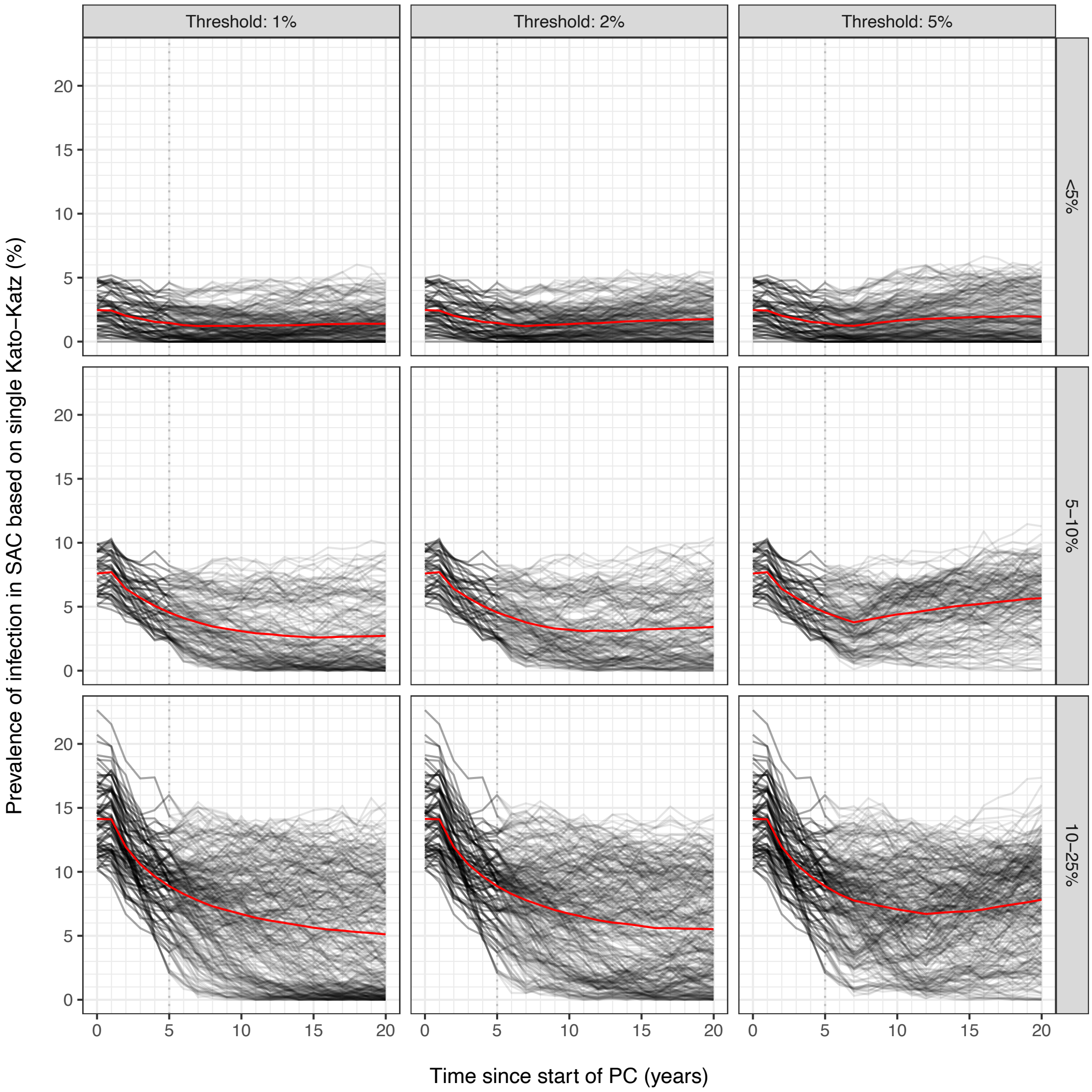


**Supplementary Figure S1. Example of model-predicted trends of *Schistosoma mansoni* infection in school age children (SAC) when using single Kato-Katz for the decision to stop preventive chemotherapy.** Black lines represent individual simulation trajectories, based on simulated surveys that took place just before each PC round. Red lines represent averages over repeated simulations. The three columns of panels pertain to different prevalence thresholds (1%, 2%, and 5%) for making the decision to stop preventive chemotherapy. The three rows of panels pertain to categories of baseline prevalence of infection in SAC (<5%, 5-10%, and 10-25%). Similar type of model predictions were produced for each of the other 7 diagnostic strategies for decisions to stop PC.

Of the 600 simulations, 546 resulted in baseline prevalences of at least 0%. Based on the baseline prevalence (i.e., prevalence at time zero), these 546 simulations were categorised into three bins: <5% (N = 171), 5-10% (N = 138), and 10-25% (N = 237). Supplementary Figure S1 provides an illustration of the model-predicted infection levels, based on a decision strategy using single Kato-Katz.

For each diagnostic strategy, decision threshold, and endemicity category, we calculated the number of person-years with heavy infection (epg ≥ 400, as measured by single KK) per 1000 capita per year and the average number of rounds PC that had been distributed (Supplementary Figure S2). As the number of PC rounds and trends in infection during the first 5 simulation years were the same for all scenarios (i.e., the first survey moment), these quantities were only calculated for the last 15 simulation years only (i.e., after the first survey moment).

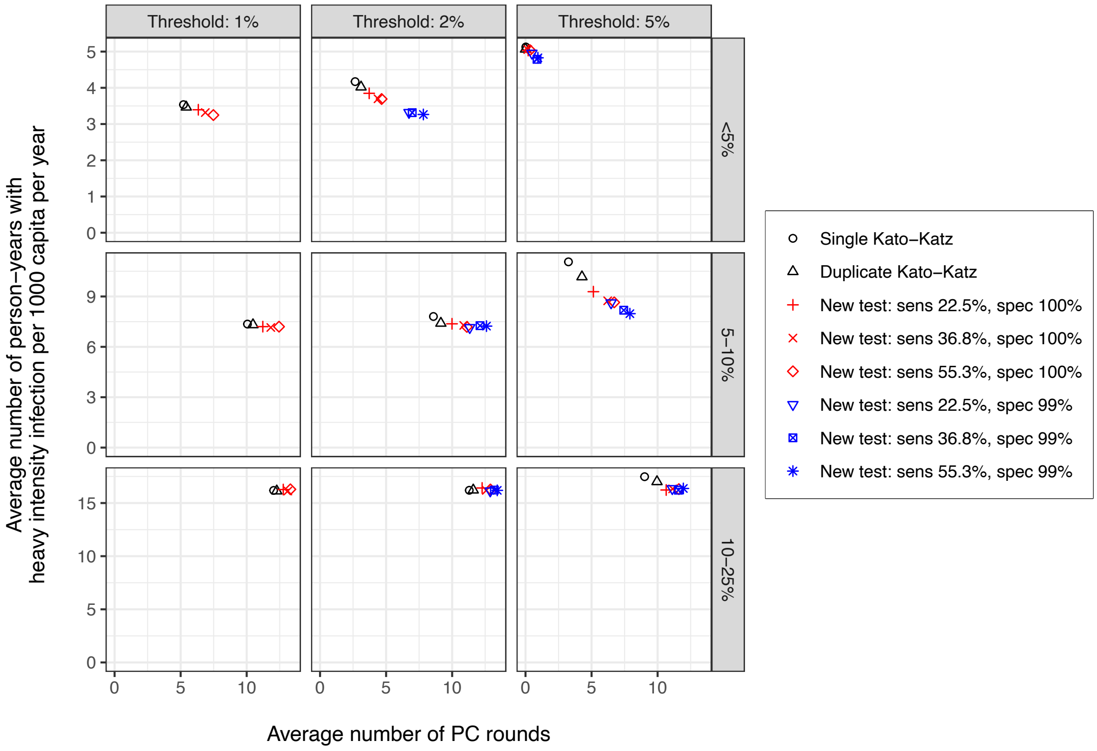


**Supplementary Figure S2. Model-predicted number of person-years with heavy *Schistosoma mansoni* infection in the general population (per 1000 capita per year) versus the average number of rounds of preventive chemotherapy (PC) targeting school age children.** Symbols and colors represent different diagnostic strategies to make decisions about stopping PC. Person-years with heavy infection and number of PC rounds were calculated only for the last 15 years of the simulation, i.e., from the point of the first survey at year 5 onwards and after the 6th PC round had taken place. This means that at most, 14 PC rounds could have been delivered after the first survey. The three columns of panels pertain to different prevalence thresholds (1%, 2%, and 5%) for making the decision to stop preventive chemotherapy. The three rows of panels pertain to categories of baseline prevalence of infection in SAC (<5%, 5-10%, and 10-25%).

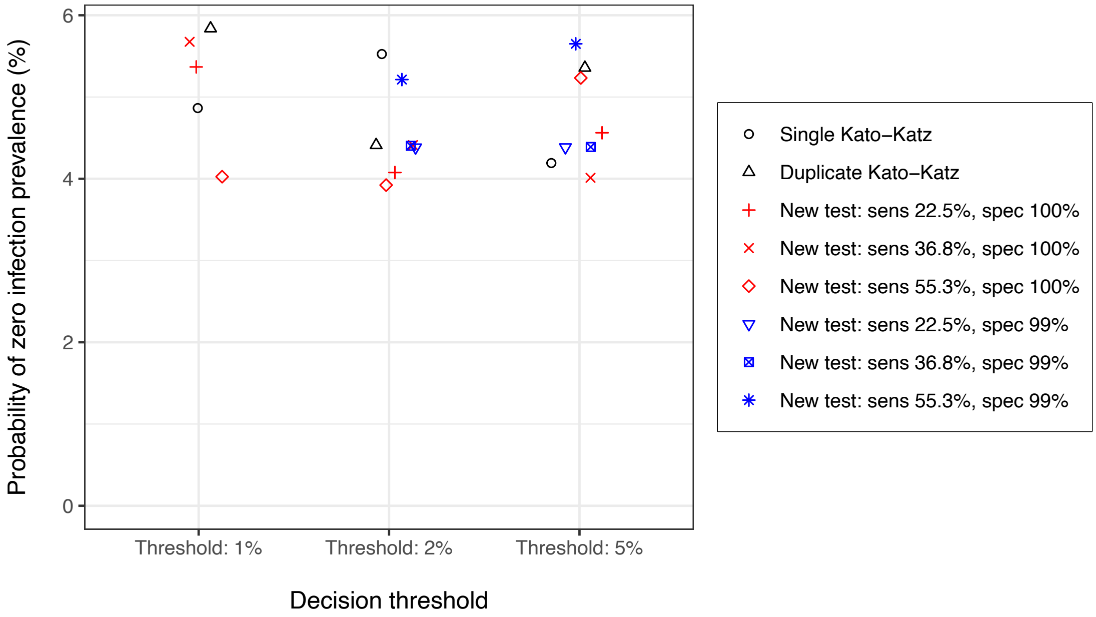


**Supplementary Figure S3. Model-predicted probability of zero infection prevalence, 20 years after the start of preventive chemotherapy (PC) targeting school age children.** Symbols and colors represent different diagnostic strategies to make decisions about stopping PC. However, for all diagnostic strategies, the probability of achieving zero infection prevalence was based simulated egg counts as measured by single Kato-Katz. Results represent a setting with baseline prevalence of infection in SAC <5% where PC was implemented at 60% coverage; for the other (higher) endemicity categories, 0% infection prevalence was never achieved with PC at 60% coverage. Probabilities were based on 171 simulations, and as such, none of the probabilities differed significantly from one another.

## References


1. Anderson RM, May RM (1985) Herd immunity to helminth infection and implications for parasite control. *Nature* **315**: 493–496.
2. Anderson RM, May RM (1991) Infectious Diseases of Humans: Dynamics and Control. Oxford & New York: Oxford University Press.
3. May RM (1977) Togetherness among Schistosomes: its effects on the dynamics of the infection. *Math Biosci* **35**: 301–343.
4. Anderson RM, May RM (1985) Helminth infections of humans: mathematical models, population dynamics, and control. *Adv Parasitol* **24**: 1–101.
5. Anderson RM, Turner HC, Farrell SH, Yang J, Truscott JE (2015) What is required in terms of mass drug administration to interrupt the transmission of schistosome parasites in regions of endemic infection? *Parasit Vectors* **8**: 553.
6. Truscott JE, Gurarie D, Alsallaq R, Toor J, Yoon N, et al. (2017) A comparison of two mathematical models of the impact of mass drug administration on the transmission and control of schistosomiasis. *Epidemics* **18**: 29–37.
7. Coffeng LE, Levecke B, Hattendorf J, Walker M, Denwood MJ (2021) Survey design to monitor drug efficacy for the control of soil-transmitted helminthiasis and schistosomiasis. *Clin Infect Dis* **72**: S195–S202.
8. de Vlas SJ, Engels D, Rabello AL, Oostburg BF, Van Lieshout L, et al. (1997) Validation of a chart to estimate true Schistosoma mansoni prevalences from simple egg counts. *Parasitology* **114 (Pt 2)**: 113–121.
9. Denwood MJ, Love S, Innocent GT, Matthews L, McKendrick IJ, et al. (2012) Quantifying the sources of variability in equine faecal egg counts: Implications for improving the utility of the method. *Vet Parasitol* **188**: 120–126.
10. Stan Development Team (2022) Stan: A C++ Library for Probability and Sampling, Version 2.21.7.
11. Plummer M (2003) JAGS: A Program for Analysis of Bayesian Graphical Models Using Gibbs Sampling. *Proceedings of the 3rd International Workshop on Distributed Statistical Computing, March 20–22,* Vienna, Austria.
12. R Development Core Team (2020) R: A Language and Environment for Statistical Computing.
